# Congenital Bilateral Ectropion in Collodion Infants: A Case Series

**DOI:** 10.7759/cureus.73437

**Published:** 2024-11-11

**Authors:** Manish K Karn, Rakhi Kusumesh, Gyan Bhaskar, Ashmita Adhikari

**Affiliations:** 1 Ophthalmology, Regional Institute of Ophthalmology, Indira Gandhi Institute of Medical Sciences, Patna, Patna, IND

**Keywords:** amniotic membrane grafting, collodion baby, congenital bilateral ectropion, exposure keratopathy, lamellar ichthyosis

## Abstract

Collodion baby is a rare congenital condition marked by a parchment-like membrane covering the body, often leading to complications such as bilateral ectropion. This condition poses risks of exposure keratopathy and other ocular issues. We present a case series of five infants with congenital bilateral ectropion associated with collodion babies, all born prematurely. Each case was treated with varying management approaches, including topical therapies and surgical interventions, with Case 1 experiencing recurrence despite surgical intervention, while the rest four cases demonstrated successful outcomes through appropriate treatments. Regular follow-up was essential for monitoring. Early and individualized treatment strategies are crucial for preventing complications in collodion babies with ectropion. This case series highlights the need for tailored management to optimize patient outcomes in this rare condition.

## Introduction

Collodion baby is an uncommon congenital condition where the entire body is covered by a tight, paper-like membrane, resembling parchment [1]. A collodion baby can manifest a variety of conditions, but it is commonly associated with lamellar ichthyosis [2]. This condition often leads to the outward turning of the eyelids (ectropion) and the eversion of the lips (eclabium). This condition is a rare autosomal recessive disorder characterized by extensive skin hyperkeratinization and occurs in approximately 1 out of every 300,000 live births [3]. Typically, newborns present with parchment-like patches that crack and peel within weeks, eventually developing into scales and scars if severe complications are avoided. Ectropion can affect one or all eyelids, particularly in lamellar ichthyosis [4]. We recently had the chance to treat such cases where both eyes of collodion babies had ectropion.

## Case presentation

Case 1

We received a referral for the management of bilateral ectropion of a three-month-old female baby with collodion syndrome who was born prematurely at 36 weeks through a cesarean section to a 30-year-old woman who had a normal pregnancy. The parents of the baby were closely related through marriage. She was the first child of her parents.

**Figure 1:**
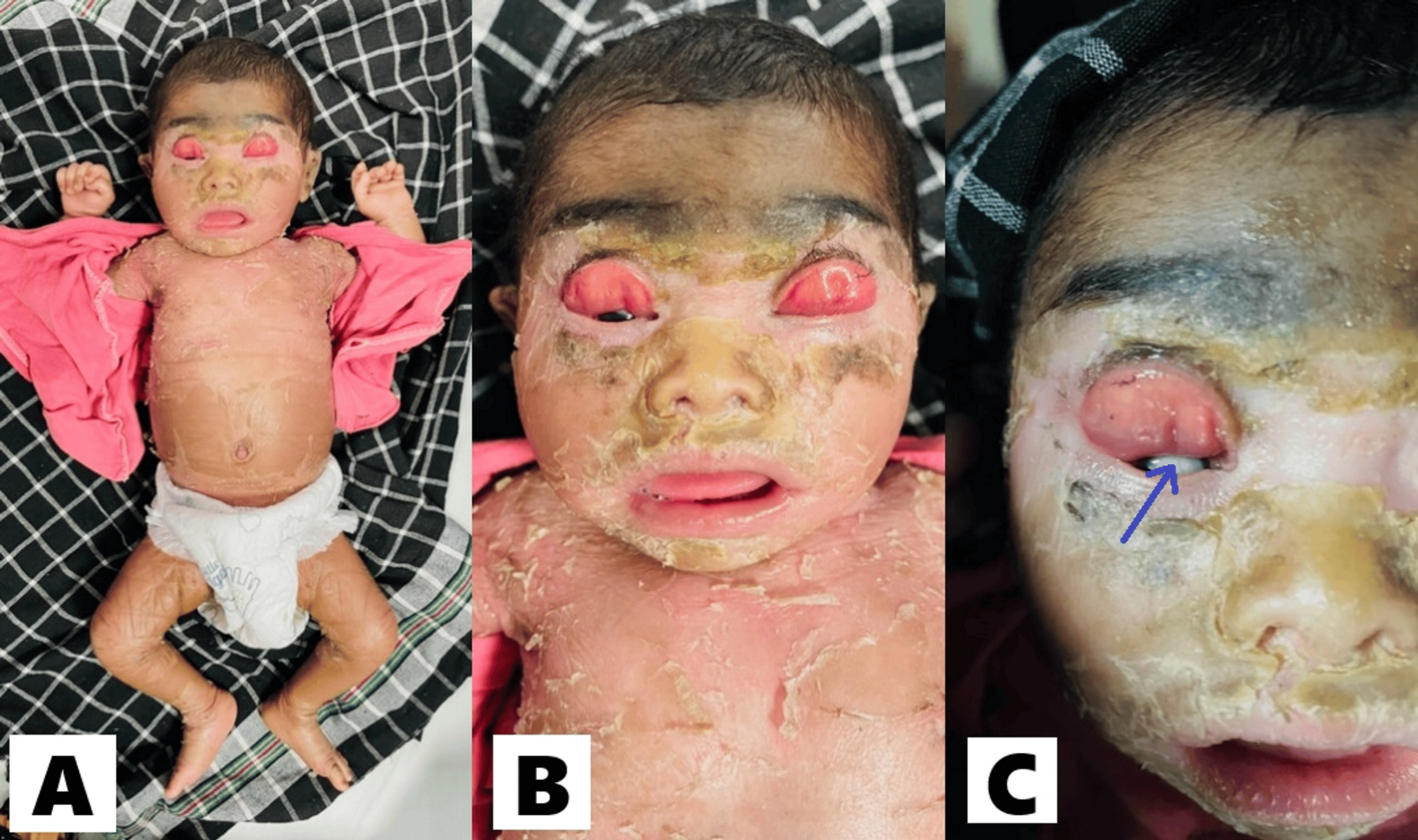
(A) Whole body covered by a tight, parchment-like membrane that is peeling off; (B) Facial features such as ectropion (eversion of eyelids) and eclabium (eversion of lips); (C) Secondary corneal ulceration due to exposure keratopathy (indicated by blue arrow).

Her entire body, including her face, was covered with a tight, parchment-like membrane and was peeling off (Figure 1A). Upon examination, we observed that the baby had outward turning of both the upper and lower eyelids, known as bilateral ectropion (Figure 1B). This condition led to the development of exposure keratopathy, a corneal problem caused by the eyelids not properly protecting the eye surface (Figure 1C). There was no sign of discharge, and no other eye abnormalities were found. Additionally, the baby had a slight turning outwards of the lips (eclabium), which caused her mouth to constantly remain open, resembling a fish. This thickened skin structure and the pulling of soft tissues around the lips and eyes led to the development of ectropion and eclabium. The baby also exhibited restricted limb movements and difficulties with suckling.

When the baby was referred to us for the treatment of ectropion, she was hemodynamically stable, and there were no signs of infection, dehydration, or compromised vital signs. The cornea was affected by exposure keratopathy resulting from the ectropion. Initially, we opted for conservative management. We administered tobramycin eye drops 0.3% every three hours, and hydroxy propyl methyl cellulose 2% eye ointment every two hours to address the eye condition. Topical emollients were recommended to soften and moisturize the skin. We advised the patient to follow up with us on a weekly basis to monitor the involvement of the cornea.

After two weeks, there was improvement, and the child was able to partially open her eyes. However, due to the tightness of the membrane, her eyes remained open, leading to secondary corneal ulceration caused by exposure. Over the course of several weeks, the shiny membrane that covered the entire body cracked and peeled off.

**Figure 2:**
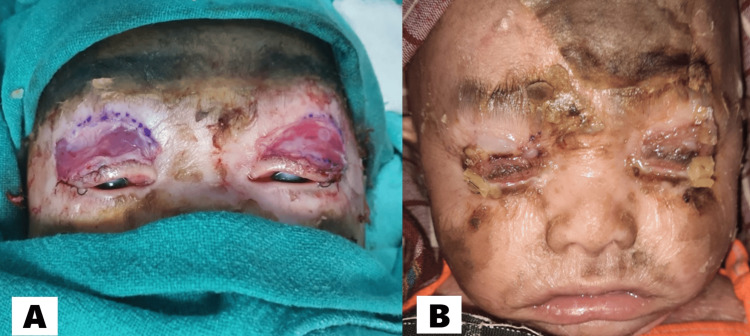
(A) Day 1 post-ectropion correction surgery; (B) Day 7 post-ectropion correction surgery.

Immediate intervention was required to manage ectropion with corneal exposure to prevent the development of corneal keratitis and opacities. Surgical intervention involves releasing the taut membrane of the upper eyelid and grafting an amniotic membrane along the tarsal plate. The graft was secured in place with fibrin glue, and lateral canthoplasty was performed to ensure proper alignment and closure of the eyelids (Figures 2A-2B). The amniotic membrane graft didn’t take up leading to scarring and recurrence of cicatricial ectropion. Resurgery was planned to correct the cicatricial ectropion.

Case 2

**Figure 3:**
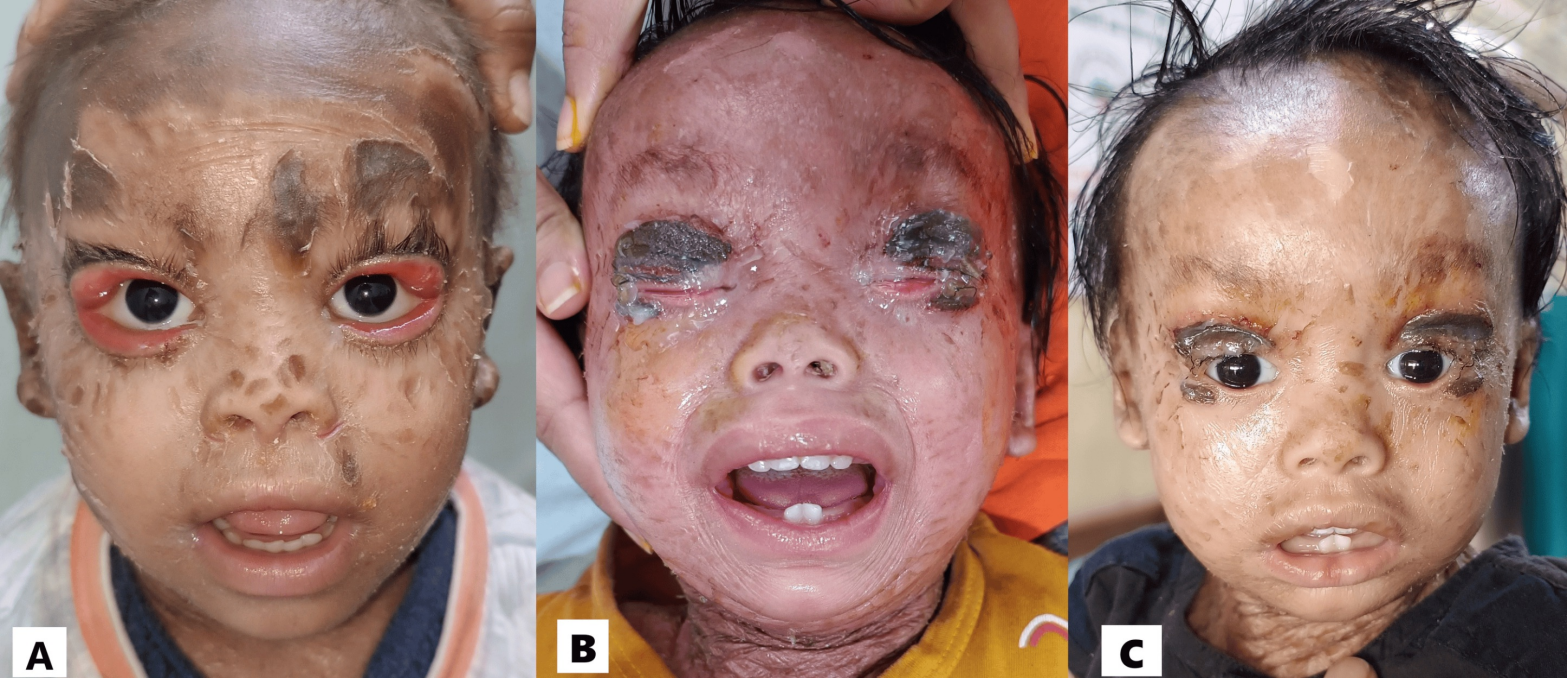
(A) Bilateral ectropion; (B) Day 1 post-ectropion correction surgery; (C) Third week post-ectropion correction surgery.

A newborn 21 days old male baby was born prematurely at 34 weeks gestation by a normal vaginal delivery to a 25-year-old mother from a non-consangious marriage. The baby was born covered in a tight, shiny, membrane-like layer, similar to cellophane. His older sibling was normal at birth. The tight skin around the eyes and mouth leads to ectropion and eclabium respectively (Figure 3A). There was no evidence of exposure keratopathy secondary to ectropion. There was no evidence of discharge. The cornea appeared to be in a healthy condition. The eyes were managed by applying methylcellulose 0.5% drops every hour and hydroxy propyl methyl cellulose 2% eye ointment every four hours. Additionally, gentle massaging of the eyelid skin with the emollients was advised. To prevent exposure keratitis, moist saline gauze was placed over the face, covering the eyes. Parents were advised for regular follow-ups. After nine months, his eyes remain open with subsequent early keratinization of the conjunctiva. Therefore, surgery to correct ectropion was scheduled due to concerns about corneal involvement and potential complications. In this instance, we opted to perform a procedure using a skin graft from the patient's mother. The plan involved releasing the tight skin over the eyelids and replacing it with the mother's skin tissue harvested from the retroauricular area during the implantation of the graft (Figure 3B). After a period of four weeks, the skin graft was successfully integrated, correcting the ectropion. His eyes now close completely with only minimal scarring (Figure 3C). He was recommended to undergo regular follow-up appointments.

Case 3

**Figure 4:**
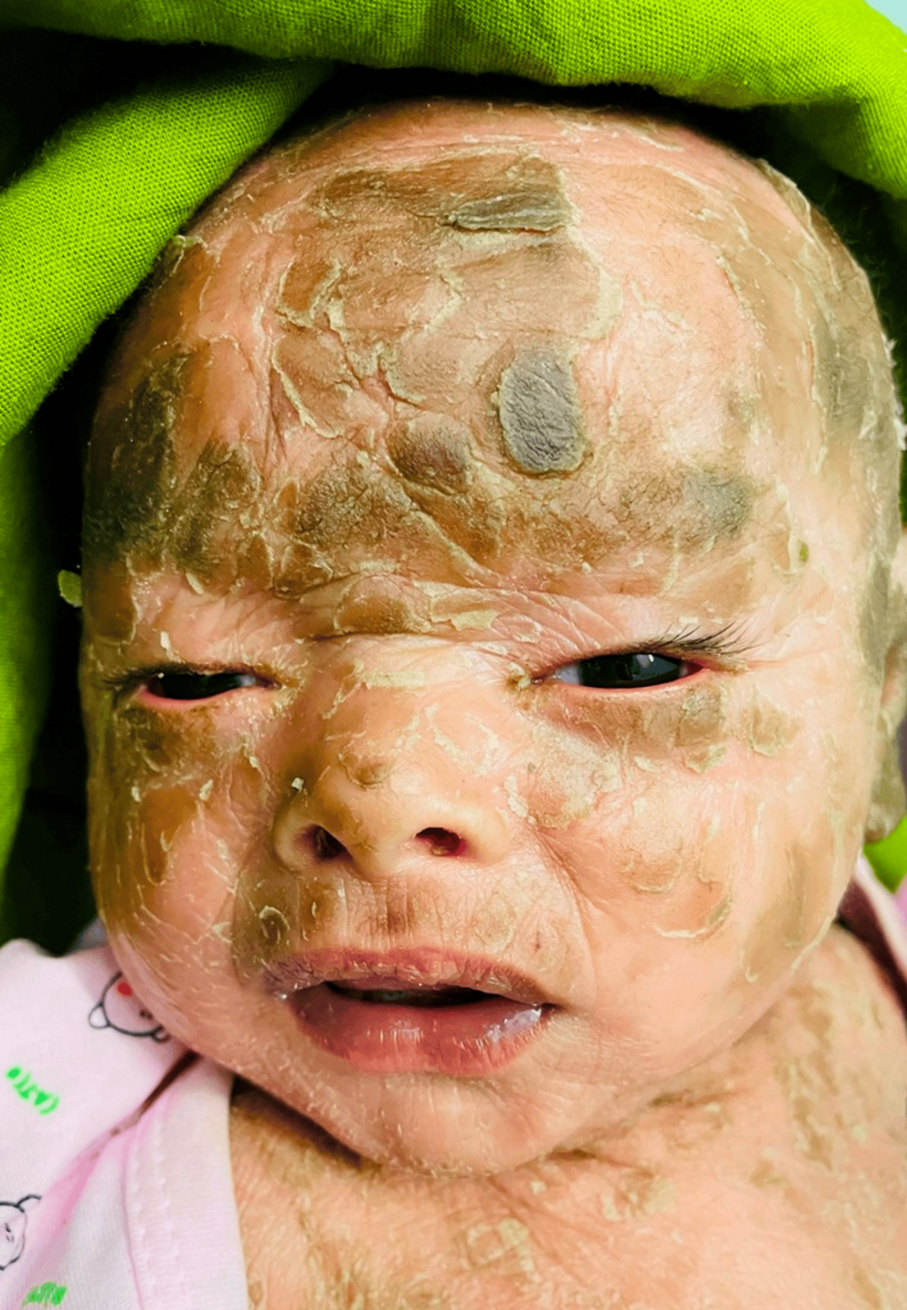
Lamellar ichthyosis with a milder presentation.

A two-month-old male baby was born prematurely at 34 weeks gestation by a normal vaginal delivery encased within a shiny, taut, cellophane-like membrane. He was the fifth born child to parents of consanguineous marriage. There is a history of similar conditions in previous births. There were no signs of exposure keratopathy resulting from ectropion, and no discharge was observed. The cornea appeared healthy (Figure 4). To manage the baby's eyes, methylcellulose 1.0% eye drops were administered every two hours, and topical emollients were recommended to soften and moisturize the skin. The patient was advised to follow-up on a weekly basis to evaluate the involvement of the cornea. After a four-week period, there was improvement, and the child was able to partially open his eyes.

Cases 4 and 5

**Figure 5:**
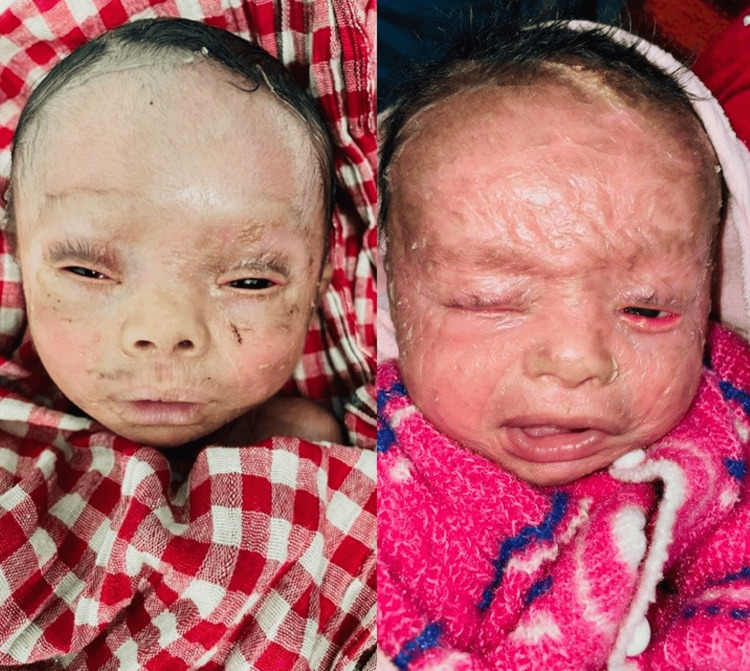
Lamellar ichthyosis in twins with a milder presentation.

Two male twin babies were born prematurely at 36 weeks via cesarean section to a 36-year-old woman who had conceived through in-vitro fertilization (IVF). Both presented with a milder form of lamellar ichthyosis. Their eyes closed partially and milder forms of ectropion were well managed conservatively with topical lubricants (carboxy methylcellulose 1.0% drops every two hours) and gentle massage of the eyelid skin with the emollients (Figure 5).

## Discussion

A collodion baby can manifest a variety of conditions, but it is commonly associated with lamellar ichthyosis [2]. Managing a collodion baby presents significant challenges due to factors such as prematurity, dehydration, temperature instability, and infection [5]. These babies are typically born prematurely and are covered in a tout, shiny membrane resembling cellophane at birth. After birth, this membrane gradually dries out and forms cracks, eventually leading to complete shedding within the initial few weeks of life [1-5]. This condition is typically caused by mutations in specific genes and is commonly associated with congenital ichthyosis, a condition characterized by scaly skin [6]. However, around 10% of collodion babies have normal skin underneath the membrane, which is a milder form referred to as "self-healing" collodion babies [7]. Congenital lamellar ichthyosis is a rare autosomal recessive disorder characterized by extensive skin hyperkeratinization. Facial features such as ectropion and eclabium (eversion of eyelids and lips) are common [7]. Arnold first described the association between lamellar ichthyosis and ectropion in 1834 [8,9]. Although ectropion occurs in 80% of cases, not all patients develop associated ocular complications such as corneal ulceration [6,8]. Notably, while upper eyelid function is compromised in lamellar ichthyosis, additional eye disorders are necessary for corneal damage to occur, potentially leading to transient or permanent visual impairment [10,11]. Contributing factors include cicatricial ectropion, lagophthalmos, abnormalities in tear production and distribution, and impaired Bell's reflex. The multifactorial nature of corneal damage is evident [12,13]. Authors differ in their criteria for surgical intervention, with some advocating for lagophthalmos as an indication, while others recommend surgery only after ocular complications arise [14-16]. Early surgical intervention can mitigate exposure keratopathy following treatment, potentially benefitting patients by preventing corneal damage.

A major challenge lies in securing viable donor sites for full-thickness skin grafts, given extensive hyperkeratotic lesions covering much of the body surface [17,18]. Ectropion recurrence post-surgery is not uncommon, often attributed to generalized skin structure abnormalities. Even grafts with normal histological structures are prone to contracture due to cicatricial processes [19]. Efforts to mitigate these risks include harvesting skin grafts larger than the original defect size. Our case reports outline the clinical presentation and strategies employed for its management (Table 1).

**Table 1:** Clinical presentations and the strategies employed for its management. * LSCS: lower segment caesarean section; ** NVD: normal vaginal delivery

Case	Age/gender/order	Consanguinity	Similar presentation in a sibling	Birth history	Management of ectropion	Outcome
1	3 months/female/singlet	+	-	LSCS* @ 36 weeks	Releasing of taut membrane and amniotic membrane grafting (AMG)	Recurrence of ectropion, resurgery needed for ectropion correction
2	3 weeks/male/singlet	-	-	NVD** @ 34 weeks	Releasing of taut membrane and skin graft taken from mother	Successful closure of palpebral fissure
3	2 months/male/singlet	++	+	NVD** @ 34 weeks	Conservative management	Ectropion corrected with medical management
4	6 weeks/male/twin	-	+	LSCS* @ 36 weeks	Conservative management	Ectropion corrected with medical management
5	6 weeks/male/twin	-	+	LSCS* @ 36 weeks	Conservative management	Ectropion corrected with medical management

## Conclusions

Typically, immediate management is prioritized for ectropion accompanied by corneal exposure due to concerns about the potential development of corneal keratitis and opacities. In these instances, utilizing a skin graft proved more effective than amniotic membrane grafting for the replacement of skin tissue after releasing the taut membrane and correcting the ectropion. In summary, based on the cases discussed, corneal damage in ichthyosis is multifactorial and not solely the result of ectropion and corneal exposure. Conservative management alone may not suffice; early surgical intervention appears crucial in preventing complications that could lead to vision impairment. Ectropion recurrence post-surgery is possible but can be managed through subsequent treatments to protect the cornea. Treatment approaches should be tailored and should be individually adjusted to every patient.
